# Ocular torsion responses to electrical vestibular stimulation in vestibular schwannoma

**DOI:** 10.1016/j.clinph.2018.08.023

**Published:** 2018-11

**Authors:** Stuart W. Mackenzie, Richard Irving, Peter Monksfield, Raghu Kumar, Attila Dezso, Raymond F. Reynolds

**Affiliations:** a University of Birmingham, School of Sport, Exercise & Rehabilitation Sciences, UK; b University Hospital Birmingham, Centre for Rare Diseases, UK

**Keywords:** Vestibular schwannoma, Asymmetry ratio, Electrical vestibular stimulation, Head impulse test

## Abstract

**Objectives:**

We determined if eye movements evoked by Electrical Vestibular Stimulation (EVS) can be used to detect vestibular dysfunction in patients with unilateral vestibular schwannoma (VS).

**Methods:**

Ocular torsion responses to monaural sinusoidal EVS currents (±2 mA, 2 Hz) were measured in 25 patients with tumours ranging in size from Koos grade 1–3. For comparative purposes we also measured postural sway response to EVS, and additionally assessed vestibular function with the lateral Head Impulse Test (HIT). Patient responses were compared to age-matched healthy control subjects.

**Results:**

Patients exhibited smaller ocular responses to ipsilesional versus contralesional EVS, and showed a larger asymmetry ratio (AR) than control subjects (19.4 vs. 3.3%, *p* < 0.05). EVS-evoked sway responses were also smaller in ipsilesional ear, but exhibited slightly more variability than the eye movement response, along with marginally lower discriminatory power (patients vs. controls: AR = 16.6 vs 2.6%, *p* < 0.05). The HIT test exhibited no significant difference between groups.

**Conclusions:**

These results demonstrate significant deficits in the ocular torsion response to EVS in VS patients.

**Significance:**

The fast, convenient and non-invasive nature of the test are well suited to clinical use.

## Highlights

• Electrical vestibular stimulation (EVS)-evoked eye movements are trackable with an infrared camera.
• Unilateral vestibular schwannoma attenuated the ocular torsion response to EVS.
• EVS-evoked ocular torsion responses provide a convenient, non-invasive vestibular assessment.

## 1 Introduction

Electrical Vestibular Stimulation (EVS) is a simple method for activating the vestibular nerve by directly applying cutaneous currents over the mastoid processes (Fitzpatrick and Day, 2004). The resulting change in vestibular afferent firing rate produces a sensation of head roll (Reynolds and Osler, 2012). This, in turn, evokes a variety of motor outputs including sway (Lund and Broberg, 1983) and orienting responses (Fitzpatrick et al., 2006). EVS also activates the vestibular-ocular reflex. The evoked eye movement is primarily torsional, with minimal lateral or vertical component (Schneider et al., 2002, Jahn et al., 2003a, Jahn et al., 2003b, Severac Cauquil et al., 2003, MacDougall et al., 2005, Mackenzie and Reynolds, 2018b).

Although EVS has mainly been used as a basic research tool, there is evidence for its clinical diagnostic potential (Dix and Hallpike, 1952). When applied in a monaural configuration, the integrity of each ear can be separately assessed. Using this approach, altered EVS-evoked responses have been reported in a variety of vestibular disorders. For example, the magnitude of ocular torsion responses are significantly reduced following intratympanic gentamicin injections (Aw et al., 2008). This has also been reported for the EVS-evoked sway response following streptomycin toxicity (Dix et al., 1949). In contrast, responses are *larger* in Meniere’s disease (Aw et al., 2013a). In a series of vestibular case studies MacDougall et al. (2005) reported systematic changes in the 3D orientation of the eye movement corresponding to specific canal deficits. These studies suggest that the EVS could supplement or even replace existing diagnostic tests. But before it can be useful as a general vestibular diagnostic, it is necessary to establish the normative and pathological responses in a variety of patients. From a practical clinical perspective, it is also desirable to develop a convenient, non-invasive and affordable version of the test for assessing the ocular response to EVS.

Here we measure the ocular response to EVS in patients with vestibular schwannoma (VS), a slow-growing benign tumour arising from the Schwann cells of the vestibulocochlear nerve. Previous research has studied EVS-evoked postural sway in VS, and compared the response to stimulation of the tumour ear to that of the healthy ear (Welgampola et al., 2013). Patients exhibit greater response asymmetry (AR) than control subjects, in terms of their standing sway response. This finding provides valuable diagnostic proof-of-principle for EVS. However, this particular postural test required patients to be capable of standing unaided on a force platform with their eyes closed and feet together. Since balance problems are a common feature of vestibular disorders, this potentially rules out a large minority of patients. In contrast, assessment of the ocular response to EVS can be performed whilst seated. Aw et al. (2013b) measured the ocular torsion response to brief pulses of square-wave EVS in four unilateral VS patients with large tumours. They reported longer response latencies as well as reduced velocity in the affected ear. Again, while this offers valuable diagnostic proof-of-principle, it is not well suited to routine clinical use due to the invasive nature of the scleral coils which were used. Here we employ a non-invasive method for recording the ocular response to sinusoidal EVS in darkness using an infrared-sensitive camera. We studied 25 unilateral VS patients with small to moderately sized tumours, and compare them to age-matched controls. Our main aim is to determine whether the patients exhibit significantly greater response asymmetry in terms of the ocular torsion response to sinusoidal electrical vestibular stimulation in each ear. We also performed two additional tests for direct comparison with the EVS ocular response; firstly, the EVS-evoked postural sway test used by Welgampola et al. (2013), and secondly, the head impulse test (HIT), since reduced HIT responses have previously reported in VS (Taylor et al., 2015, Tranter-Entwistle et al., 2016). The results show that our EVS-evoked ocular torsion test out-performed the HIT test in terms of discriminatory power and was marginally better than the postural sway test, while being more convenient.

## 2 Methods

### 2.1 Participants

25 patients (9 male) aged 30 to 80 (mean ± SD; 61 ± 13 years) were recruited from University Hospital Birmingham. The presence of a vestibular schwannoma (VS) was diagnosed by magnetic resonance imaging and quantified using the maximum extrameatal tumour diameter (Kanzaki et al., 2003). 17 healthy controls (9 males) aged 40–80 (mean ± SD: 68 ± 8 years) with no known neurological or vestibular disorder were studied for the purpose of collecting normative data in a healthy population. All participants gave informed written consent to participate. The experiment was approved by South Birmingham Research Ethics Committee and performed in accordance with the Declaration of Helsinki. Patient’s tumour measurements and symptoms are presented in Table 1.

**Table 1:** Patient Tumour characteristics and symptoms.

ID	VS side	Location	Tumour type	PTA (dB)	SDS (%)	ICL (mm)	ICD (mm)	Koos Grade	HL	TIN	BD
1	R	IAC/CPA	Solid	50	53	18.2	16.4	2	+	+	−
2	L	IAC	Solid	23	100	9	6.4	1	+	+	+
3	L	IAC/CPA	Solid	48		14.3	10.2	2	+	+	+
4	L	IAC/CPA	Solid	47	60	20.7	16.3	2	+	+	+
5	R	IAC	Cystic	30		10	6	1	+	+	−
6	L	IAC/CPA	Solid	58	20	11.5	13.3	2	+	+	+
7	R	IAC/CPA	Solid	17	87	15.6	12.3	2	+	+	+
8	R	IAC/CPA	Solid	53		20.4	15	2	+	+	+
9	R	IAC/CPA	Solid	3	100	16.2	10.2	2	−	−	+
10	L	IAC/CPA	Solid	23	86	7.5	5.1	1	+	+	−
11	L	IAC	Solid	8	97	4.1	4.3	1	−	−	+
12	L	IAC/CPA	Solid	30	98	8	6	1	+	+	+
13	R	IAC/CPA	Solid	23		17.1	12.2	2	+	+	−
14	L	IAC	Solid	75	40	2.5	4	1	+	+	+
15	L	IAC/CPA	Solid	67	17	20	16	2	+	−	+
16	R	IAC/CPA	Solid	43	90	16	10.9	2	+	−	+
17	L	IAC/CPA	Solid	15	100	22	12.4	2	+	−	+
18	R	IAC/CPA	Solid	7	30	19.7	10.9	2	+	+	+
19	L	IAC/CPA	Solid	50	73	15.7	6.7	1	+	+	+
20	L	IAC/CPA	Solid	35	70	20	18.7	2	+	+	−
21	R	IAC/CPA	Cystic	75	60	16	11.5	2	+	+	+
22	R	IAC/CPA	Solid	37	70	19.3	10.3	2	+	+	+
23	L	IAC/CPA	Solid	30	90	33	35.4	3	+	−	−
24	L	IAC/CPA	Solid	60		27.3	17.1	2	+	+	+
25	R	IAC/CPA	Cystic	72	42	37.8	16.8	2	+	−	−

R = Right, L = Left; IAC = Internal auditory canal, CPA = Cerebellopontine angle; PTA = Pure Tone Average; SDS = Speech Discrimination Score; ICL = intracanalicular length; ICD = intracanalicular diameter; HL = Hearing loss; TIN = Tinnitus; BD = Balance Disturbance, + symptomatic, − non-symptomatic.

### 2.2 Evaluating tumour size

**Fig. 1:**
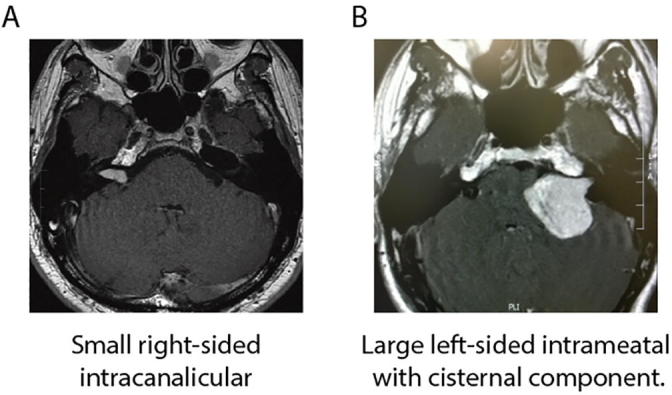
MRI scan of vestibular schwannoma. (A) A patient with a small right-sided intracanalicular tumour. (B) A patient with a large left-sided intrameatal tumour with a cisternal component.

Koos classification and internal acoustic canal filling were assessed by MRI. Koos classification is a four-point grading system based on the size of the tumour (intracanalicular and cisternal) G1 < 1 cm, G2 1–2 cm, G3 2–3 cm, G4 > 3 cm (Koos et al., 1998). Fig. 1A depicts a small right-sided intracanalicular tumour while Fig. 1B depicts a large left-sided intrameatal tumour with a cisternal component. Most participants were classified as Koos grade 2, which is partially attributable to the treatment procedure, whereby anyone with a tumour over 2 cm in diameter is offered cyberKnife, ultimately resulting in their exclusion from the study.

### 2.3 EVS-evoked torsional eye movements

*Protocol –* We used the protocol described by Mackenzie and Reynolds (2018b); “Participants were seated with the head restrained (SR Research Ltd. Ontario, Canada) for the duration of each 10 s stimulation period. Prior to each trial participants were instructed to focus on the lens of an infrared camera and not to blink before being immersed into darkness. An invisible infrared light (940 nm) was used to illuminate the eye during each trial. No fixation light was provided to ensure that any horizontal and vertical eye movements were not suppressed”.

*Electrical Vestibular Stimulation –* Electrical vestibular stimulation (EVS) (2 Hz, peak ± 2 mA) was delivered using carbon rubber electrodes (46 × 37 mm) in a monaural cathodal or anodal configuration. Four electrodes were coated in conductive gel, two were secured to the mastoid processes and two overlying the C7 spinous process using adhesive tape. Stimuli were delivered from an isolation constant-current stimulator (AM Systems, Carlsberg, WA, USA). Four conditions (2 sides × 2 polarities) were repeated 3 times giving a total of 12 trials.

**Fig. 2:**
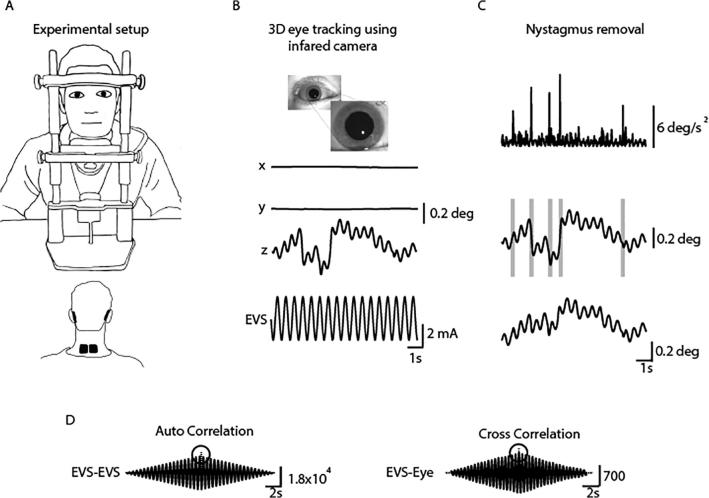
Analysis of EVS-evoked ocular responses. Adapted from Mackenzie and Reynolds (2018b). (A) Subjects sat in darkness with the head fixed during 10 s EVS stimuli (2 Hz, ±2 mA), delivered in a monaural configuration. (B) 3D eye movements were recorded using an infrared camera and then tracked off-line. (C) An eye acceleration threshold was used to detect fast phase movements which were then removed using a compensatory inverse nystagmus algorithm. (D) Response gain was determined by the ration of the peak EVE-eye cross correlation to the peak EVE-EVS auto correlation.

*Data Acquisition and Analysis –* We used the same analysis described by Mackenzie and Reynolds (2018b) (Fig. 2); “Torsional eye movements were sampled at 50 Hz using an infrared camera (Grasshopper 3, Point Grey Research Inc, Richmond, BC, Canada). Eye movements were tracked and quantified off-line using a commercially available planar tracking software (Mocha Pro V5, Imagineer Systems Ltd. Guildford, UK). Torsional motion was tracked using iris striations. This technique has previously been validated across stimulation frequency range of 0.05–20 Hz (Mackenzie and Reynolds, 2018b). Nystagmus fast phases were automatically identified and removed (Mackenzie and Reynolds, 2018b). The magnitude of the eye response was measured as the peak value of the stimulus-response cross-correlation. Gain was then calculated by dividing this value by the peak stimulus autocorrelation to normalise with respect to the input stimulus”. An asymmetry ratio was then calculated from the gains of both ears.

### 2.4 EVS evoked postural adjustments

**Fig. 3:**
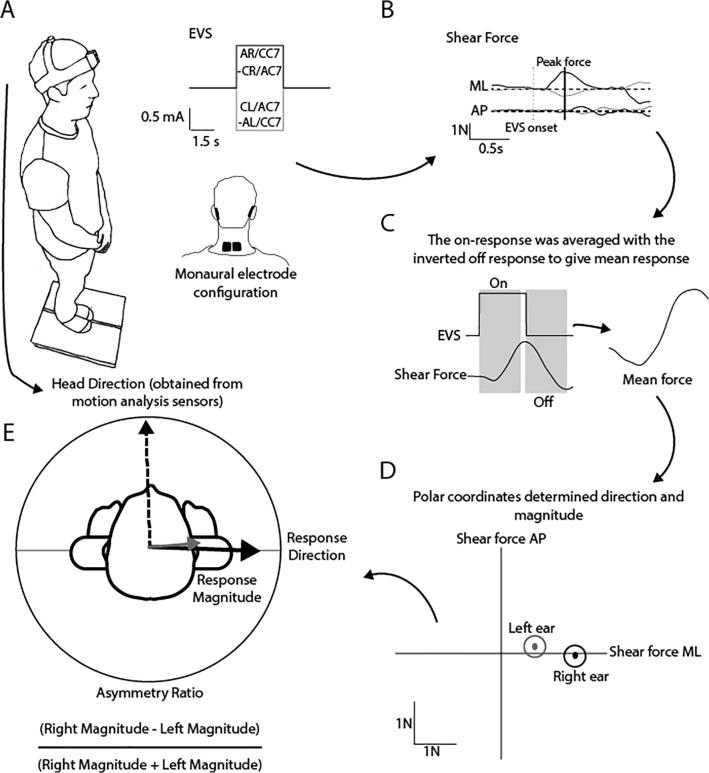
EVS-evoked postural sway experimental setup. (A) Participants stood on a force platform while receiving monaural EVS stimuli. (B) Ground-reaction forces were used to determine response direction and magnitude. For the left ear, anodal responses were inverted and cathodal for the right. (C) The EVS off response was inverted and averaged with the on response. (D) and (E) The magnitude and direction (atan Fx/Fy) of the peak force vector within this time window was measured from a participant average. An asymmetry ratio was calculated using the left and right ear response magnitudes.

*Protocol -* Participants stood in the centre of a force plate, without shoes, with feet together and hands held relaxed in front of them for the duration of each 60 s stimulation period (Fig. 3A). Prior to each trial participants were instructed to face a visual target at eye level, 1 m in front of them before closing their eyes for the duration of the trial.

*Electrical Vestibular Stimulation –* EVS was delivered in a monaural configuration to evoke postural sway. EVS was applied in sequences of six 3 s impulses of 1 mA, separated by a 6 s gap. The side of the active electrode (left or right) and the polarity (cathode or anode) was randomised across trials. Two sides and two polarities gave a total of 4 conditions (Anode-Left/Cathode-C7, Anode-Right/Cathode-C7, Cathode-Left/Anode-C7 and Cathode-Right/Anode-C7). Four repeats of each condition resulted in a total of 24 impulses per condition (96 in total).

*Data Acquisition and Analysis -* Head position was sampled at 50 Hz in the form of Euler angles using a Fastrak sensor (Polhemus Inc, Colchester, Vermont, USA) attached to a welding helmet frame worn by the participants. As previously described by (Mackenzie and Reynolds, 2018a); “any offset in yaw or roll angle between head orientation and sensor orientation was measured using a second sensor attached to a stereotactic frame, and subsequently subtracted. A slight head up pitch position was maintained throughout each trail to ensure Reid’s plane (line between inferior orbit and external auditory meatus) was horizontal, ensuring an optimal response to the virtual signal of roll evoked by vestibular stimulation (Fitzpatrick and Day, 2004). The evoked sway response to vestibular stimulation was recorded in the form of ground reaction forces at 1 kHz using a Kistler 9281B force platform (Kistler Instrumente AG, CH-8408 Winterthur, Switzerland)”.

Analysis of EVS-evoked shear force is depicted in Fig. 3. Similar analysis techniques to Welgampola et al. (2013) were used. To increase signal-to-noise ratio of the response, the averages to the two stimulation polarities were combined separately for the mediolateral (Fx) and anteroposterior (Fy) direction. As the two polarities evoked responses in opposite directions, one polarity was inverted before the averaging process took place. For the left ear, the anodal response was inverted where as for the right ear the cathodal response was inverted, this was to ensure both ears resulted in a direction response towards the right. The ‘off’ response to stimulus cessation was combined with the ‘on’ response to stimulus onset. Again, the on and off responses are oppositely directed, hence the off response was inverted prior to the averaging process. The force response was quantified as the peak force vector between 200–800 ms after stimulus on/offset. The magnitude and direction (atan Fx/Fy) of the peak force vector within this time window was measured from a participant average. An asymmetry ratio from stimulation of each ear was calculated using the equation in Fig. 3E, where R and L represent right and left magnitude respectively.

### 2.5 Head impulse test (HIT)

*Protocol –* Participants received 20 (10 right, 10 left) impulses while seated. HIT involves a small (∼30°), rapid (50–300°/s) head rotation in yaw, evoked by the experimenter. Participants were instructed to fixate on a visual target located 1 m in front of them throughout the HIT.

**Fig. 4:**
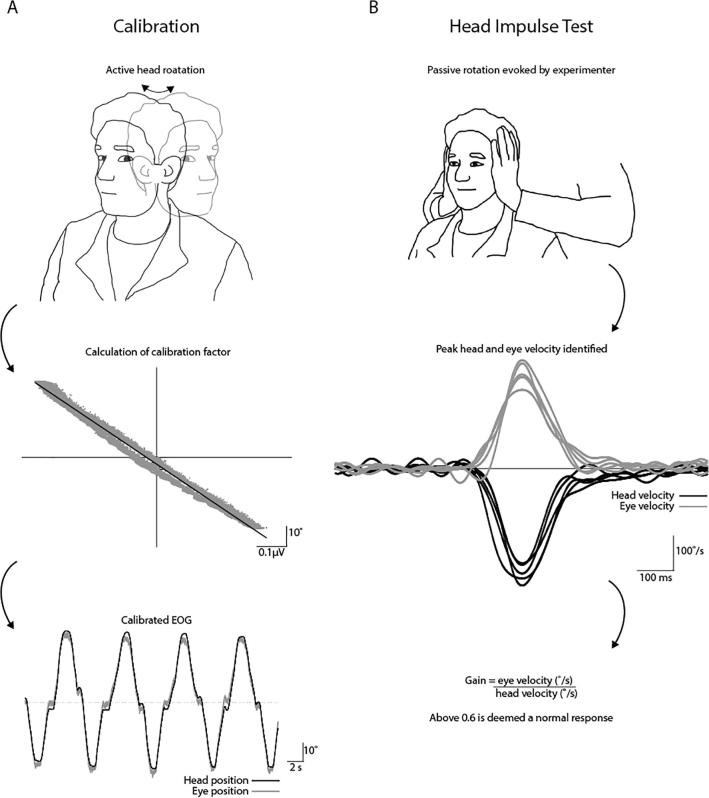
Head Impulse Test. (A) EOG and head position, recorded during active yaw rotation, were plotted against each other to derive a calibration factor for EOG. (B) The experimenter performed multiple HITs towards the left and right ears. Peak velocity of the head and eye were used to calculate gain.

*Calibration –* Eye kinematics were recorded using electro-oculography (EOG), thus requiring conversion from µV to degrees of rotation. This was achieved by having the participants rotate the head in yaw while keeping the eyes fixated on a target, allowing a regression to be calculated between EOG and degrees of head rotation, measured using a motion tracker (Fig. 4A). The calculated calibration was used to calibrate all subsequent EOG signals into degrees. The success of this calibration process can be observed in Fig. 4A, where head position (black trace) and inverted eye position (grey trace) closely match each other.

*Data Acquisition and Analysis -* Eye kinematics were sampled at 1 kHz using EOG. Two non-polarizable skin electrodes were applied near the outer canthi and a reference electrode to the forehead. Prior to electrode placement the skin was prepared by rubbing the skin with an abrasive electrode gel, all excess gel was removed before the area of skin was cleaned with an alcohol wipe and left to dry. The calibrated eye position for each head impulse was low pass filtered using a 5th order Butterworth (cut-off 10 Hz), from which eye velocity could be calculated. Head position was sampled at 50 Hz in the form of Euler angles using a Fastrak sensor attached to a welding helmet frame worn by the participants (Polhemus Inc, Colchester, Vermont, USA). Head velocity during the HIT was sampled at 1 kHz using a gyro sensor located on the welding helmet worn by the participant. Offline analysis of the data was automated using MATLAB software. Peak head velocity and peak eye velocity were automatically selected and used to determine the horizontal gain (eye velocity/head velocity). A gain of 0.68 or greater was deemed normal (MacDougall et al., 2009). An asymmetry ratio (AR) was calculated for each participant.

### 2.6 Statistical analysis

To detect if the patients healthy ear was indeed healthy, it was compared to a random selection of right and left ear responses from the control group using an independent *t* test (SPSS). Response gain (unitless) was used to quantify both HIT and EVS-evoked torsional eye movements, whereas peak force (N) was used to quantify the magnitude of the EVS-evoked. An unpaired *t* test was used to compare asymmetry ratios between controls and patients. We also performed correlations between EVS-evoked postural AR’s and EVS-evoked eye movement AR’s. A correlation between tumour size and AR was also performed. Pearson correlations were used to determine significance.

For all statistical tests, significance was set at *p* < 0.05. Means and standard deviations are presented in text and figures, unless otherwise stated*.*

## 3 Results

### 3.1 EVS-evoked eye movement

**Fig. 5:**
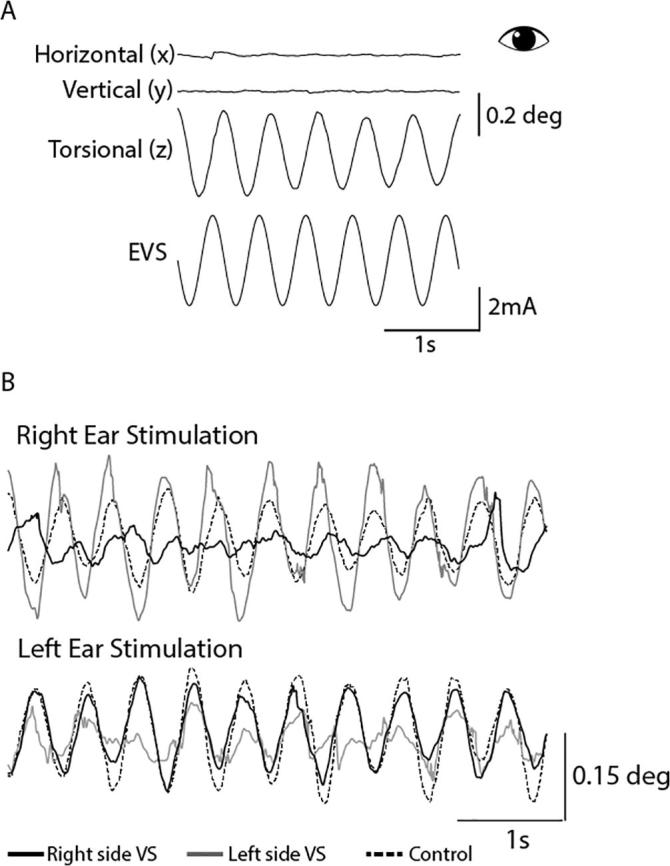
3D eye movements evoked by EVS stimulation. (A) A representative control participant. EVS induces a sensation of head roll about the naso-occipital axis. This leads to the torsional (z) eye movements being much larger than both the horizontal (x) and vertical (y) components of the eye movements. For this reason, only torsional eye movements were analysed. (B) A healthy individual (black dashed trace) shows a similar response gain when either the right or left ear is stimulated. However, the vestibular schwannoma patients show a reduced response magnitude during ipsilesional stimulation (solid black and grey traces).

Sinusoidal EVS evoked a strong torsional eye movement, with minimal horizontal or vertical components (Fig. 5A) (Mackenzie and Reynolds, 2018b). Therefore, only torsional eye movements were used in subsequent analysis. Fig. 5B depicts torsional eye position in two schwannoma patients (one left and one right-sided VS) and a control subject. The control subject showed similar responses to left and right ear stimulation. Both patients showed attenuated responses during ipsilateral stimulation.

As reported in Mackenzie and Reynolds (2018b), there was a ∼90°phase lag between the stimulus and response, with no difference between groups, or between contralesional and ipsilesional stimulation.

**Fig. 6:**
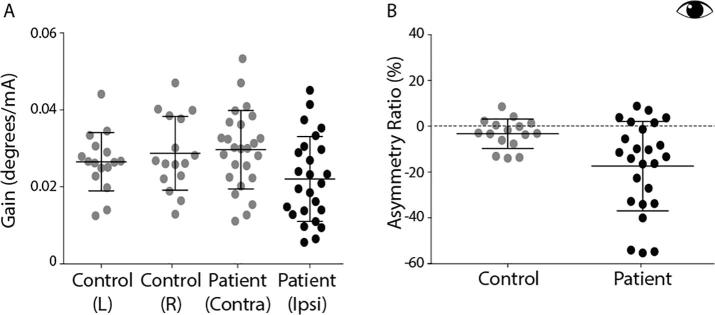
EVS-evoked torsional eye movement response magnitudes and asymmetry ratios. (A) Response gains for control’s left and right ear stimulation and patient’s contralesional ear (grey) and patients ipsilesional ear (black). (B) Asymmetry ratio for controls (grey) and patients (black). Mean and SD presented.

Response gain is illustrated in Fig. 6A. Control subjects exhibited equal gain for left and right ear stimulation. Contralesional stimulation in patients produced similar values to the control group (*T*_(55)_ = 0.41, *p* > 0.05). However, ipsilesional stimulation produced an attenuated response. This is apparent in the asymmetry ratios, where the mean values were −3.27% and −19.38% for controls and patients, respectively (Fig. 6B, *T*_(48)_  =2.53, *p* < 0.05).

### 3.2 EVS-evoked postural responses

**Fig. 7:**
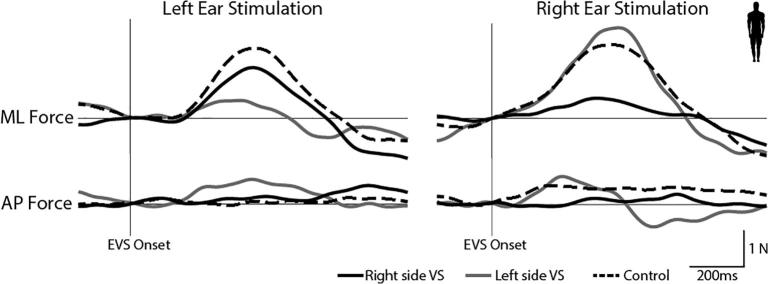
EVS-evoked sway response. EVS during a head forward (0°) orientation produces a compensatory sway response as shown by a force increase in the ML force. A healthy individual (black dashed trace) shows a similar response magnitude when either the right or left ear is stimulated. However, the vestibular schwannoma patients show a reduced response magnitude during ipsilesional stimulation (solid black and grey traces).

Fig. 7 depicts EVS-evoked ground reaction forces in two schwannoma patients (one left and one right-sided VS) and a control subject standing face-forward. EVS primarily evoked a mediolateral force response, with minimal anterior-posterior response. The control subject showed very similar responses to left and right ear stimulation. In contrast, both patients showed markedly attenuated responses during ipsilesional stimulation.

**Fig. 8:**
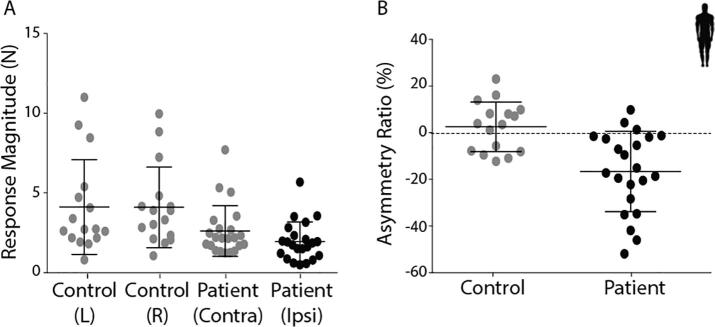
EVS-evoked postural response magnitudes and asymmetry ratios. (A) Response magnitude for controls left and right ear stimulation and patients contralesional ear (grey) and patient ipsilesional ear stimulation (black). (B) Asymmetry ratio for controls (grey) and patients (black). Mean and SD presented.

In control subjects, peak force responses were similar for left and right ear stimulation (Fig. 8A). In patients, while stimulation of the contralesional ear produced similar responses to control subjects (*T*_(42)_ = 1.85, *p* > 0.05), ipsilesional forces were attenuated. This was confirmed by a significant difference in asymmetry ratio between the two groups (Fig. 8B; Controls = 2.6%, patients = −16.6%; *T*_(36)_ = 3.92, *p* < 0.05).

**Fig. 9:**
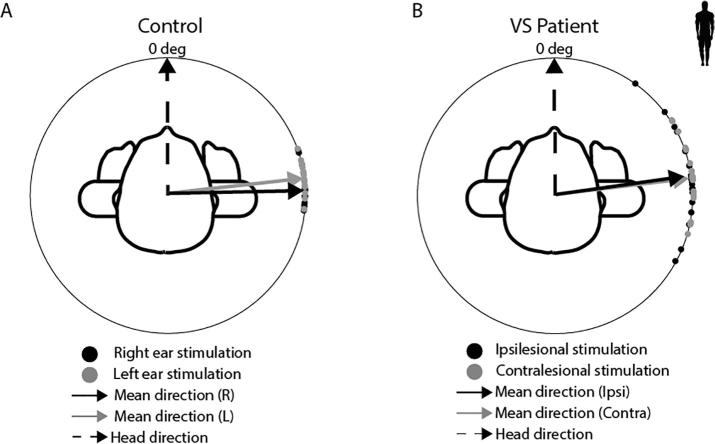
EVS-evoked postural response direction. (A) Controls produced a mean force response (solid arrows) directed 90° to head orientation (dashed arrow) for both left (grey) and right (black) ear stimulation. (B) Patients produced the same response direction as controls for both contralesional (grey) and ipsilesional (black) stimulation. Anode-left and cathode-right trials have been flipped in direction to match anode-right and cathode-left.

In addition to measuring the magnitude of the EVS-evoked force vector, we also measured its direction (Fig. 9). With the head facing forwards, anodal EVS over the right ear evoked a postural response directed along the inter-aural axis. Schwannoma had no effect upon the direction of this response, with all controls and patients responses oriented in the same direction (F_(4,96)_ = 2.13, *p* > 0.05).

### 3.3 HIT-evoked eye movement responses

**Fig. 10:**
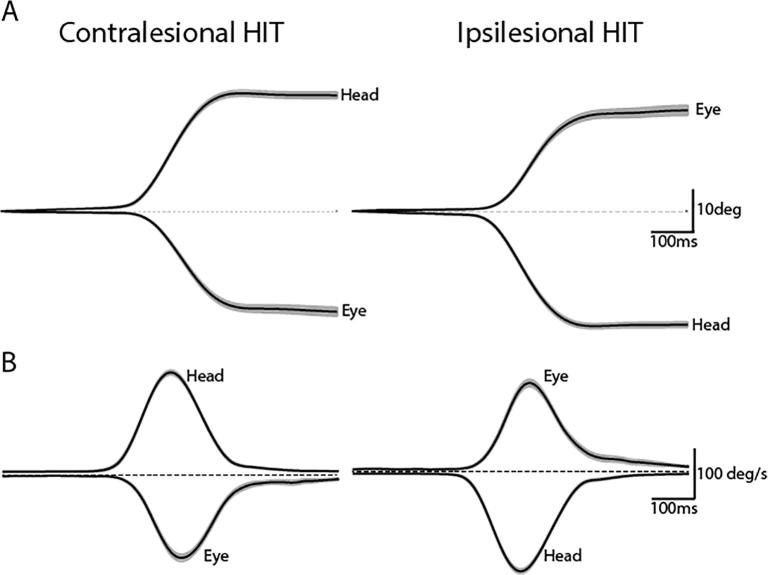
HIT amplitude and velocity. (A) Mean amplitudes of 28 and 27° rotation were achieved for contralesional and ipsilesional HITs respectively. (B) Mean velocities of 197 and 200°/s were produced during contralesional and ipsilesional HITs respectively. These values are all within the range of a successful HIT. Mean (black trace) and 95% confidence limits (grey shaded region) are presented.

Mean head and eye kinematics during the HIT test are shown in Fig. 10 for schwannoma patients. Mean head rotation amplitude (and peak velocity) was 28° (197°/s) and 27° (197°/s) for contralesional and ipsilesional directions, respectively.

**Fig. 11:**
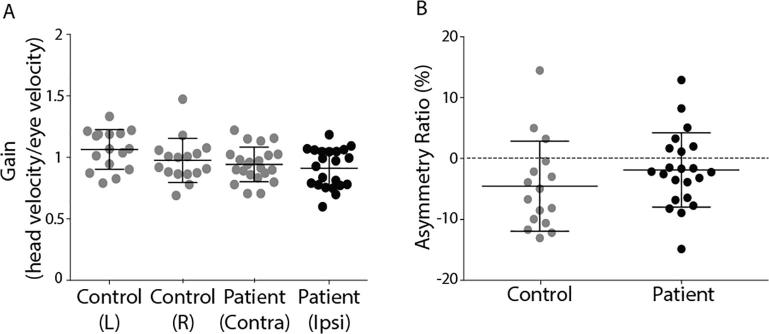
HIT Response gains and asymmetry ratios. (A) HITs in healthy (towards left or right ear) and VS patients (contralesional or ipsilesional) resulted in response gains of ∼1. (B) Asymmetry ratios. Mean and SD are presented, along with individual subject data.

Gain values (eye/head velocity) were approximately 1 in both patients and control subjects, irrespective of head direction (Fig. 11A). There was no difference in the asymmetry ratio between the patient and control groups (*T*_(36)_ = 1.29, *p* = 0.41).

### 3.4 Comparison of ocular and postural responses to EVS in schwannoma patients

**Fig. 12:**
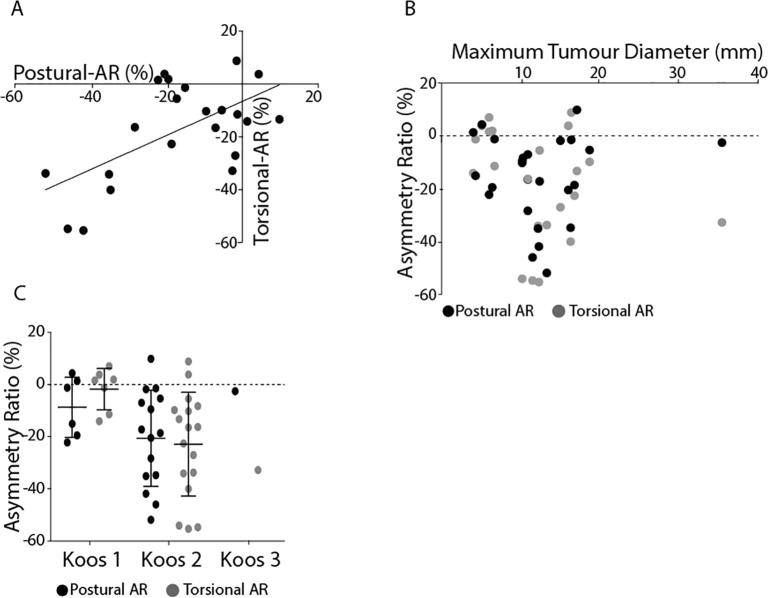
Experimental comparisons. (A) Both posture and eye movement tests produced similar asymmetry ratios, resulting in a significant positive correlation. (B) Neither postural nor eye movement asymmetry ratios showed any correlation with tumour diameter. (C) Patients were grouped according to Koos classification (measure of tumour size). Postural asymmetry ratios did not differ between classifications, whereas torsional evoked asymmetry ratios showed a significant increase from Koos grade 1–2.

Fig. 12A shows the ocular and postural asymmetry ratios plotted against each other for the patient group. The two methods exhibited a moderate correlation (*r* = 0.60, *p* < 0.05). Neither ocular nor postural asymmetry exhibited any significant relationship with tumour size (Fig. 12B). However, when patients were classified according to their Koos grade, those with Koos 1 showed smaller ocular asymmetry than Koos 2 (*T*_(22)_ = 2.69, *p* < 0.05). There was no effect of Koos grade upon the postural asymmetry ratio (*T*_(19)_ = 1.46, *p* > 0.05).

## 4 Discussion

We measured the ocular torsion response to sinusoidal electrical vestibular stimulation (EVS) using the same stimulation and recording techniques described in Mackenzie and Reynolds (2018b). The only significant modification was the use of a monaural rather than binaural stimulus, so that each ear could be assessed separately. When we applied this technique to vestibular schwannoma patients we found that the ocular response was significantly reduced in the ipsilesional versus contralesional ear. When combined with the speed, comfort and practicality of the technique, this establishes the potential utility of the EVS-evoked eye movement as a clinical diagnostic test.

Mean ocular response asymmetry ratio in the VS patients was ∼20%, being significantly greater than that of control subjects. This was also true for the EVS-evoked postural response. This is broadly consistent with Aw et al. (2013), who observed a ∼50% reduction in the ocular torsion velocity in the affected ear of VS patients. However, the limited sample size (four patients), different stimulation and recording techniques, and unreported tumour size limits comparison to the current findings. In our data there was considerable overlap between patients and controls for both the ocular and postural tests. This contrasts with the results of Welgampola et al. (2013). They measured the ground reaction force response to EVS in the same way as described here, and found ∼40% asymmetry in the patient response, and zero overlap with control subjects. However, tumour size in their patient group was more than double that here (27 vs. 12 mm). Therefore, the difference is probably related to the extent of vestibular nerve damage in the two patient cohorts. So although our test is not suitable for discriminating early-stage Schwannoma patients from control subjects (∼50% sensitivity; Fig. 5B), the variability within our patient group likely reflects genuine differences in vestibular function.

The asymmetry in the patient ocular response correlated with that of their postural response, suggesting that both results reflect the extent of the underlying vestibular deficit caused by the tumour. The magnitude of EVS-evoked sway responses are affected by numerous factors including head orientation, biomechanics, proprioceptive acuity and baseline sway (Pastor et al., 1993, Fitzpatrick and McCloskey, 1994, Fitzpatrick and Day, 2004, Mian and Day, 2009). The EVS-evoked eye movement is simpler by comparison, consisting of a tri-neuronal sensorimotor arc combined with the minimal inertia of the eyeball. Hence, the ocular response theoretically constitutes a less variable test of vestibular function. Indeed, we did observe less variability in the ocular asymmetry of control subjects compared to their postural response (6.4 vs 10.7% AR). But perhaps more important than subtle differences in diagnostic efficacy between the two tests is the large difference in practicality. The eye movement recording was performed over a ∼10 min period in seated subjects. It is readily applied to patients with a high degree of postural instability and/or physical disability. Indeed, two patients were unable to complete our postural test, while all undertook the ocular recordings. Furthermore, the use of infrared video offers a practical alternative to invasive techniques such as scleral coils or marking the sclera with a surgical pen to aid tracking.

Patients with Koos grade 2 tumours exhibited greater mean asymmetry than those in the smaller grade 1 category, but there was no correlation between tumour size and asymmetry ratio for either test. This tallies with Welgampola et al. (2013) whose data showed no correlation between EVS-evoked force and tumour size in eight patients with tumours spanning 17–40 mm (see Table 1 from Welgampola et al, 2013). The lack of a systematic relationship between tumour size and vestibular deficit is perhaps unsurprising, since limited or absent correlations have also been shown for hearing loss (Nadol et al., 1996, Mahmud et al., 2003), although this may not be true for much larger tumours (Schuknecht, 1974). Our data also exhibited no relationship between tumour diameter and hearing loss or speech discrimination (see Table 1). This absence of a size effect is likely due to the non-uniform manner in which tumour growth impinges upon the auditory-vestibular nerve.

In addition to measuring EVS-evoked postural sway magnitude we also determined sway direction, and found this to be normal in the patient group. Furthermore, the phase lag between the EVS stimulus and the ocular response was also normal. These findings suggest that sensorimotor transformation processing for vestibular information is entirely normal in VS patients. It is simply the magnitude of the responses which are affected.

In contrast to previous reports, gain values for our HIT test were ∼1 for all subjects and directions, with no significant asymmetry in the VS patients, nor any difference between patients and controls. Tranter-Entwistle et al. (2016) reported mean gains of 0.73 and 0.90 during the horizontal canal video HIT test (vHIT) for the ipsilesional and contralesional side, respectively, with 10 of their 30 patients exhibiting < 0.79 (ipsi) gain. Similarly, Taylor et al. (2015) reported vHIT gains of 0.75 (ipsi) and 0.9 (contra) for the horizontal canal. Potential reasons for the null HIT response here might be differences in head movement kinematics, recording techniques and patient tumour location or size. Regarding kinematics, our peak head displacement (velocity) was ∼27° (200°/s), being within most accepted range values for a valid HIT test (Jorns-Haderli et al. (2007): 20–40° (∼300°/s), MacDougall et al. (2009): 5–20° (50–250°/s), Taylor et al. (2015): 10–20° (50–300°/s), McGarvie et al. (2015): (100-200°/s), Tranter-Entwistle et al. (2016): (>150°/s)). Regarding technique, we used electro-oculography (EOG) rather than video for recording lateral eye movements. Although EOG has slightly poorer resolution than video (∼1 versus 0.5 deg), it is not immediately obvious how this would affect gain. Furthermore, any systematic change in gain caused by such technical differences would affect both directions equally so would not influence asymmetry. Regarding tumour location, VS can arise from the superior or inferior branch of the vestibular nerve (Khrais et al., 2008). Since the horizontal canal is innervated by the superior branch, a normal HIT test might occur if damage is restricted to the inferior branch. Consistent with this, most studies do indeed show that the superior branch is less commonly affected in VS (Khrais et al. (2008): 76% single nerve involvement with 91.4% inferior and 6% superior, 24% >1 nerve, via surgical identification. Ylikoski et al. (1978): 80% superior, 20% inferior via caloric test. Clemis et al. (1986): 50% superior via auditory tests. Komatsuzaki and Tsunoda (2001): 84.8% inferior, 8.9% superior via surgical identification). However, this still does not account for the positive results of Taylor et al., 2015, Tranter-Entwistle et al., 2016 for the horizontal canal. Regarding tumour size, this was 19 mm in Taylor et al. (2015) and ∼7–13 mm in Tranter-Entwistle et al. (2016) which is similar to, or slightly greater than our mean value of 12 mm. Hence it is not immediately apparent why our VS patients exhibited normal HIT gains, but it raises the possibility that the EVS-evoked ocular response is a more sensitive measure of vestibular deficiencies than HIT. Further comparative studies in a larger variety of vestibular disorders are needed to confirm this.

The diagnostic utility of the EVS-evoked ocular response across a broader range of vestibular disorders may depend upon its precise site of action. While not established beyond doubt, EVS currents most likely alter neural firing rate via the spike trigger zone of the primary afferent (Goldberg et al., 1984, Goldberg, 2000, Fitzpatrick and Day, 2004). This implies that the EVS response can only reveal deficits downstream of the hair cell. Vestibular schwannoma certainly constitutes such a deficit, which explains the impaired responses seen here. However, it has also been reported that gentamicin-induced vestibular toxicity impairs EVS-evoked eye movements (Aw et al., 2008). Since acute gentamicin toxicity kills vestibular hair cells, this could be interpreted as evidence that EVS stimulates the hair cell rather than the primary afferent. However, vestibular afferents have a high resting firing rate, and loss of hair cell input may conceivably reduce their firing rate and/or their excitability. Such a loss of excitability could diminish the response to an externally applied current, analogous to a drop in spinal excitability presenting as a diminished H-reflex (Baldissera et al., 2001). But irrespective of the precise mechanism of action, the evidence of gentamicin-induced deficits in the EVS-evoked response provides encouraging evidence that it could diagnose peripheral as well as central vestibular deficits, at least if such deficits affect hair cell function. To establish the precise diagnostic scope of EVS requires a direct comparison against established tests, such as caloric irrigation, vestibular evoked myogenic potential and chair rotation, in a wider group of vestibular disorders.

In summary, we have demonstrated that EVS-evoked eye movements can be recorded in a fast, convenient and non-invasive fashion in order to detect asymmetries in vestibular function. Further work is required to validate this technique against existing tests such as caloric irrigation, and in a wider group of vestibular pathologies.

## Conflicts of interest

None of the authors have potential conflicts of interest to be disclosed.
